# Hepatopancreatoduodenectomy with delayed division of the pancreatic parenchyma when utilizing a right lateral approach to the superior mesenteric artery

**DOI:** 10.1186/s40792-024-01965-z

**Published:** 2024-07-02

**Authors:** Aoi Hayasaki, Naohisa Kuriyama, Benson Kaluba, Tatsuya Sakamoto, Haruna Komatsubara, Koki Maeda, Toru Shinkai, Daisuke Noguchi, Takahiro Ito, Kazuyuki Gyoten, Takehiro Fujii, Yusuke Iizawa, Akihiro Tanemura, Yasuhiro Murata, Masashi Kishiwada, Mitsunaga Narushima, Shugo Mizuno

**Affiliations:** 1 Department of Hepato-Biliary-Pancreatic and Transplant Surgery, Mie University School of Medicine, 2-174 Edobashi, Tsu, Mie 514-8507 Japan; 2 Department of Plastic and Reconstructive Surgery, Mie University, Tsu, Mie Japan

**Keywords:** Hepatopancreatoduodenectomy, Delayed division of the pancreatic parenchyma, Right lateral approach, Transhepatic hilar approach, Perihilar cholangiocarcinoma

## Abstract

**Background:**

Hepatopancreatoduodenectomy (HPD) is a high-risk surgical procedure. Delayed division of the pancreatic parenchyma (DDPP) was reported as a novel technique in HPD for reducing postoperative pancreatic fistula. However, it is often difficult to dissect the pancreatic head nerve plexus while leaving the pancreatic parenchyma intact, particularly in patients with a bulky tumor with vascular invasion. Of the various reported approaches to the superior mesenteric artery, the right lateral approach can provide a useful surgical field to conduct DDPP in HPD.

**Case presentation:**

A 78-year-old man visited a local clinic with itching and jaundice. Laboratory tests revealed elevated hepatobiliary enzyme, total bilirubin, and tumor markers. Enhanced computed tomography, endoscopic retrograde cholangiopancreatography, and intraductal ultrasonography of the bile duct were performed, and he was diagnosed with perihilar cholangiocarcinoma with invasion to the right hepatic artery (40 × 15 mm, Bismuth IIIa, cT3N0M0 cStage III). After neoadjuvant chemotherapy, he underwent left hepatectomy with caudate lobectomy, pancreatoduodenectomy, and combined resection of right hepatic artery using DDPP with a right lateral approach to the superior mesenteric artery. The pathological diagnosis was perihilar cholangiocarcinoma ypT3N1M0 ypStage IIIC, R0 resection. He was discharged on postoperative day 57 in good health and has been doing well for 6 months since the surgery.

**Conclusions:**

We present an effective application of the right lateral approach to the superior mesenteric artery in DDPP during HPD. This procedure can provide a clear surgical field to easily divide the pancreatic head nerve plexus before transection of the pancreatic parenchyma.

## Background

Hepatopancreatoduodenectomy (HPD) was first introduced in 1980 by Takasaki et al. for the treatment of locally advanced gallbladder cancer [1]. The indication of HPD for biliary cancer remains controversial because of its high mortality (0 − 18%) and morbidity (33 − 82%) [2–5]. In particular, the incidence of postoperative pancreatic fistula (POPF) after HPD was reported as 19.7 − 89.5% [3, 5–7]. To reduce the risk of POPF, Chiba et al. and Sugiura et al. introduced novel HPD techniques involving pancreatic parenchyma transection-delayed approach and delayed division of the pancreatic parenchyma (DDPP), respectively [8, 9]. Meanwhile, minimally invasive pancreatoduodenectomy is now widely performed, and various approaches to the superior mesenteric artery (SMA) have been reported [10–14]. Of these, Ninomiya et al. introduced the right lateral approach that combined the right and posterior approaches in robot-assisted pancreatoduodenectomy [15].

Herein, we present a patient with perihilar cholangiocarcinoma who received HPD with DDPP using a right lateral approach to the SMA.

## Case presentation

A 78-year-old man visited a local clinic with itching and jaundice. His medical history included distal gastrectomy and Billroth I reconstruction for duodenal ulcer. Abdominal ultrasonography showed dilatation of the intrahepatic bile duct. Laboratory tests revealed elevated hepatobiliary enzymes (aspartate aminotransferase 62 U/L, alanine aminotransferase 106 U/L, alkaline phosphatase 296 U/L), total bilirubin (13.8 mg/dL), and tumor markers (carcinoembryonic antigen 9.6 ng/mL, carbohydrate antigen 19–9 172.2 U/mL).

**Fig. 1:**
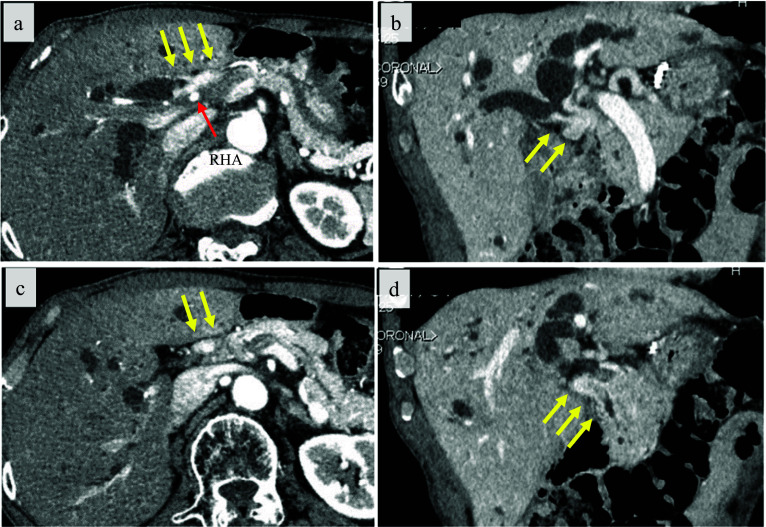
Enhanced computed tomography showed circumferential wall-thickening at the common hepatic duct (yellow arrow) with suspected invasion into the right hepatic artery (RHA; red arrow)

**Fig. 2:**
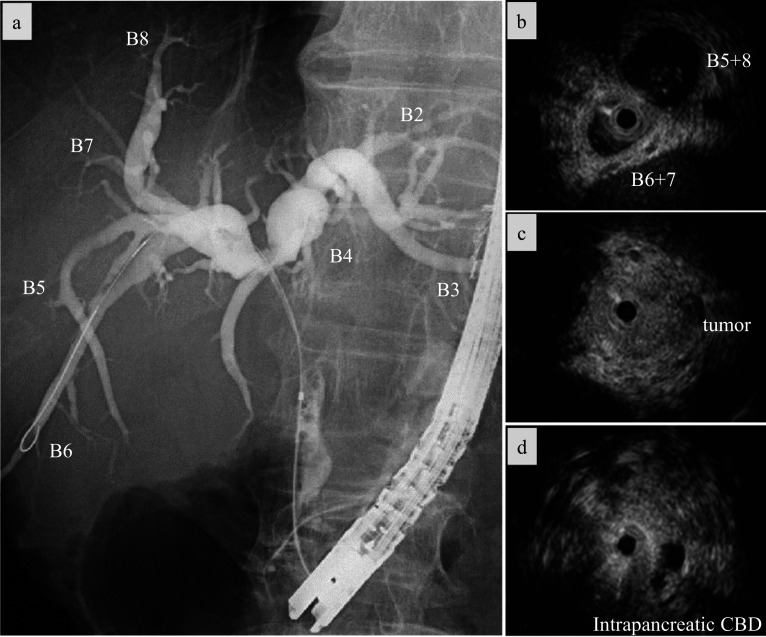
Assessment of bile duct. **a** Endoscopic retrograde cholangiopancreatography showed stenosis of the common hepatic duct. **b − d** Intraductal ultrasonography revealed bile duct wall-thickening from just below the B4 bifurcation and at the anterior–posterior bifurcation to the intrapancreatic common bile duct (CBD). B5 + 8, right anterior bile duct; B6 + 7, right posterior bile duct

Enhanced computed tomography showed circumferential wall-thickening of the bile duct from the confluence of the right and left bile ducts to the intrapancreatic bile duct, with suspected invasion of the right hepatic artery (RHA) (Fig. 1a − d). Endoscopic retrograde cholangiopancreatography was performed, and the bile duct biopsies were positive at the tumor and negative at the B2/3 and B4 bifurcations (Fig. 2a). Intraductal ultrasonography of the bile duct revealed thickening of the bile duct wall from just below the B4 bifurcation and at the anterior − posterior bifurcation to the intrapancreatic bile duct (Fig. 2b − d). Based on these findings, the patient was diagnosed with perihilar cholangiocarcinoma (40 × 15 mm, Bismuth IIIa, cT3N0M0 cStage III) [16]. The indocyanine green retention rate at 15 min was 25%, with a clearance rate of 0.093. According to our institutional protocol, neoadjuvant chemotherapy using gemcitabine, cisplatin, and S-1 was started. After chemotherapy, tumor markers decreased (carcinoembryonic antigen 4.4 ng/mL, carbohydrate antigen 19–9 30.1 U/mL) and the tumor size shrunk (35 × 15 mm, ycT2aN0M0 ycStage II).

**Fig. 3:**
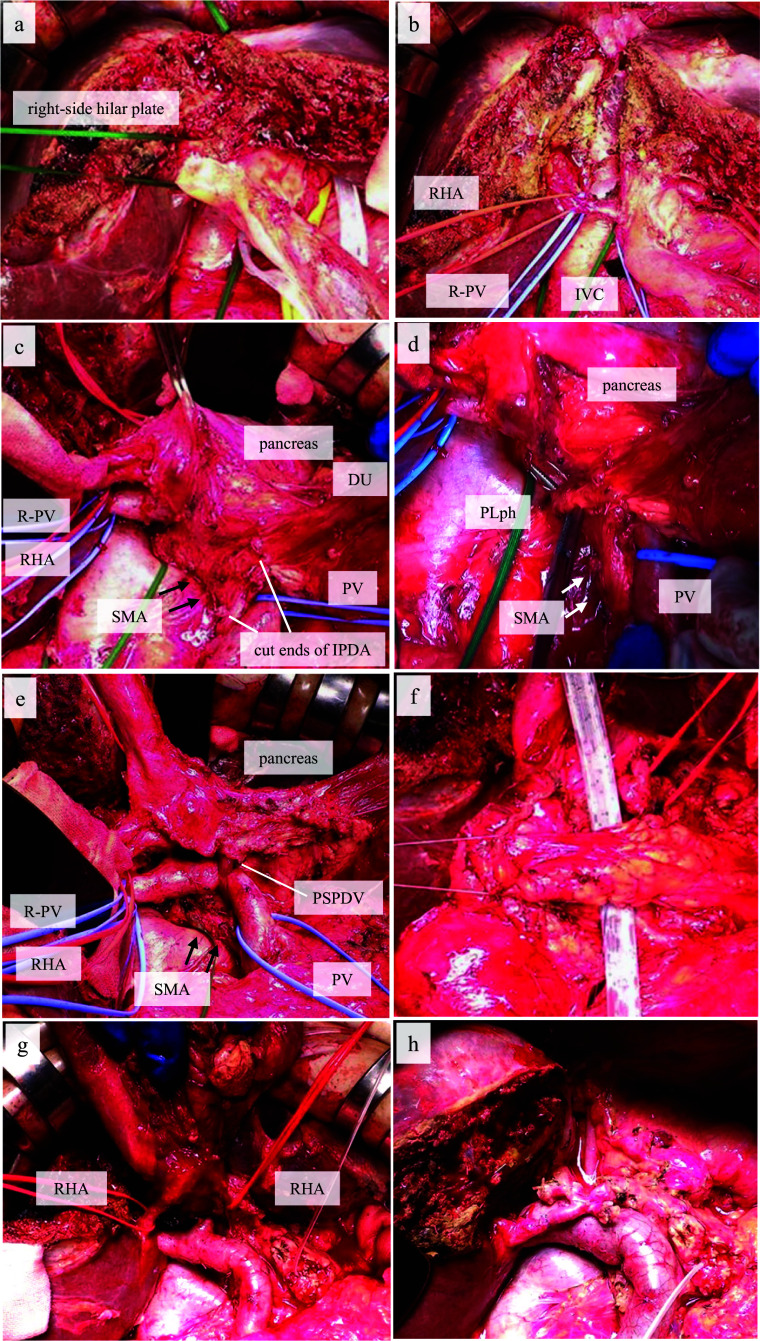
Procedural description of hepatopancreatoduodenectomy using the right lateral view in addition to the transhepatic hilar approach. **a** The right-side hilar plate was taped. **b** The right hepatic duct was first cut according to the transhepatic hilar approach. **c**, **d** The pancreaticoduodenum was rotated clockwise, and the mesopancreas and the pancreatic head nerve plexus (PLph) were divided from the right lateral view. **e** The posterior superior pancreaticoduodenal vein (PSPDV) was divided. **f** The pancreatic parenchyma was transected. **g** Macroscopic tumor involvement of the right hepatic artery (RHA). **h** Hepatopancreatoduodenectomy with combined resection of the RHA was performed. *R-PV* right branch of the portal vein, *SMA* superior mesenteric artery, *DU* duodenum

The patient underwent left hepatectomy with caudate lobectomy, pancreatoduodenectomy, and combined RHA resection using DDPP with a right lateral approach to the SMA. After hepatic mobilization, we first transected the hepatic parenchyma and confirmed the negative margin of the proximal bile duct according to the transhepatic hilar approach [17] (Fig. 3a, b), pancreatoduodenectomy using a right lateral view was started, and a Kocher maneuver was performed. The Treitz ligament was partially detached to obtain mobility of the pancreatic head. The pancreaticoduodenum was rotated clockwise to the left side, and the mesopancreas and pancreatic head nerve plexus (PLph) were divided along the right side of the SMA from the right lateral view, with division of the inferior pancreaticoduodenal artery and the posterior superior pancreaticoduodenal vein (Fig. 3c–e). Finally, the pancreatic parenchyma was transected (Fig. 3f). Because tumor involvement of RHA was observed intraoperatively, combined resection of the RHA was performed and the HPD was completed. The RHA was microscopically reconstructed (Fig. 3g, h). We performed a hepaticojejunostomy between the three bile duct orifices (B5 + 8, B6, B7) and the single jejunum orifice. Next, pancreaticojejunostomy using a duct-to-mucosa and modified Blumgart anastomosis, gastrojejunostomy, and Braun anastomosis were performed. The operative time was 626 min and the blood loss was 505 g.

The pathological diagnosis was perihilar cholangiocarcinoma ypT3N1M0 ypStage IIIC, R0 resection. A 27 × 14 × 55 mm area of moderately differentiated adenocarcinoma was observed from the right hepatic duct to the distal bile duct, which invaded the peribiliary fat tissue and the pancreatic parenchyma.

Postoperatively, the patient developed bacteremia from cholangitis, POPF (biochemical leakage), a delayed empty stomach, and a thrombus in the middle hepatic vein to the inferior vena cava, which recovered with conservative treatment such as management of drains, antibiotic therapy, and anticoagulants. He was discharged on postoperative day 57 in good health, although the hospital stay was longer because of the delayed empty stomach. He has been doing well for 6 months since the surgery.

## Discussion

Our procedure of HPD with DDPP utilizing right lateral approach to the SMA is useful and practical, and allowed us to easily dissect the PLph before transection of the pancreas. Sugiura et al. reported an anterior approach to the SMA for PLph dissection in DDPP, although they also stated that it was extremely difficult to dissect the plexus while leaving the pancreatic parenchyma in patients with vascular invasion at the perihilar region or at the distal bile duct [9].

Of the various SMA approaches [10–15], surgeons choose the optimal approach for each patient based on their tumor location, tumor size, vascular invasion, and other factors. We believe that the application of a right lateral approach in DDPP can solve the difficulty to dissect the plexus while leaving the pancreatic parenchyma reported by Sugiura et al. Moreover, in the right lateral approach [15], a Kocher maneuver was performed, the Treitz ligament was partially detached to obtain mobility of the pancreatic head, and then the pancreaticoduodenum was rotated clockwise to the left side. This approach allows a wider view of the SMA in the long axis direction and a safer approach to the SMA compared to the posterior approach. The patients who need HPD for wide-spreading bile duct cancer often have preoperative cholangitis or repeated stent replacements, resulting in tissue sclerosis and adhesions around the bile duct. So, this approach is useful because it allows a dorsal approach with less inflammation. In addition, this view may be useful in cases with bulky tumor, replacement RHA from SMA, and severe post-endoscopic retrograde cholangiopancreatography pancreatitis in the pancreatic head. In our case, the pancreatic head had rotated slightly to the left due to previous distal gastrectomy and Billroth I reconstruction, so we thought this view would be a safer approach. As described above, although it has also disadvantage in the difficulty of recognizing the dissection from the posterior view, the right lateral approach is one useful approach to conduct DDPP. It is important to know the various approaches to SMA and choose the appropriate approach for the patient's situation in order to perform this high-risk procedure safely.

In our institute, we perform HPD using the transhepatic hilar approach, in which transection of the hepatic bile duct is performed to confirm a cancer-negative margin early in the operation [17]. This approach provides a wide surgical view and enables the surgeon to confirm the resectability and possibility of reconstruction of the major blood vessels. The transection of the bile duct should be last to gain margin in oncological aspect, however, perihilar cholangiocarcinoma is often associated with proximal and distal extensive horizontal tumor spread. Preoperative step biopsy recognizes the extent of the tumor, but it may be difficult to assess because of epithelial changes due to biliary stent replacement, or it may be negative in the epithelium but positive in the epithelial or extraepithelial wall. Thus, in rare cases, proximal margins are positive with invasive carcinoma even when the liver is resected to the limit of remnant liver volume and function. Since HPD has a very high mortality and morbidity rate, we think that in such cases, liver resection only, without PD, resulting in positive proximal and distal margins with invasive carcinoma may be a choice, considering the overall balance of other factors, such as the patient's general condition.

Improvements to our procedure are required given the loss of time for vascular reconstruction and hepaticojejunostomy. In the present patient, it took approximately 2 h for hepatic artery reconstruction and 1 h for hepaticojejunostomy. It took time for the microscopic setting and additional resection of the RHA due to intraoperative heat-induced intimal damage. Careful manipulation without applying heat to the vessel and improvement of the technique of vascular reconstruction are essential. Generally, we suggest that vascular reconstruction is performed after the specimen is resected because the reconstructed vessels can easily become twisted, bent, or hyperextended depending on their placement. Furthermore, pancreatojejunostomy is usually made after hepaticojejunostomy because the hepatic ducts are often multiple, small, and located at the bottom of the hepatic transection plane. Note that it is possible to prevent some saponification by placing a tube in the main pancreatic duct and draining the pancreatic juice outside of the surgical field during the vascular reconstruction.

## Conclusion

We presented an effective application of the right lateral approach to the SMA in DDPP during HPD. This procedure can provide a clear surgical field to easily divide the PLph and may reduce the incidence of POPF by shortening the interval between pancreatic transection and reconstruction.

## Data Availability

All data supporting the conclusions of this article are included within the published article and the accompanying images.

## Abbreviations

HPD: Hepatopancreatoduodenectomy
DDPP: Delayed division of the pancreatic parenchyma
POPF: Postoperative pancreatic fistula
SMA: Superior mesenteric artery
CT: Computed tomography
RHA: Right hepatic artery
PLph: Pancreatic head nerve plexus
